# Optimizing Postoperative Glucose Management in CABG Patients: Exploring Early Transition to Subcutaneous Insulin

**DOI:** 10.3390/jcdd11110348

**Published:** 2024-11-01

**Authors:** Hamza Alzghoul, Joel Weimer, Abigail Antigua, Geran Maule, Mohamed F. Ismail, Ward Althunibat, Raju Reddy, Abdul Ahad Khan, Nehan Sher, Robyn Meadows, Akram Khan

**Affiliations:** 1 Graduate Medical Education, University of Central Florida College of Medicine, Orlando, FL 32816, USA; hamza.alzghoul@hcahealthcare.com (H.A.); geran.maule@hcahealthcare.com (G.M.); mohamed.ismail@hcahealthcare.com (M.F.I.); ward.althunibat@hcahealthcare.com (W.A.); 2 Department of Pharmacy, HCA Florida North Florida Hospital, Gainesville, FL 32605, USA; joel.weimer@hcahealthcare.com (J.W.); abigail.antigua@hcahealthcare.com (A.A.); 3 Division of Pulmonary and Critical Care, Department of Medicine, University of Texas, Austin, TX 78712, USA; raju.reddy@austin.utexas.edu; 4 Division of Pulmonary, Critical Care and Sleep Medicine, University of Arizona College of Medicine-Phoenix, Banner University Medical Center, Phoenix, AZ 85006, USA; mirahadk@gmail.com (A.A.K.); nehan.sher2@bannerhealth.com (N.S.); 5 Graduate Medical Education, HCA Healthcare, Brentwood, TN 37027, USA; robyn.meadows@hcahealthcare.com; 6 Division of Pulmonary, Allergy and Critical Care Medicine, Department of Medicine, Oregon Health and Science University, Portland, OR 97239, USA

**Keywords:** coronary artery bypass, insulin, glycemic control, postoperative care, diabetes mellitus

## Abstract

Introduction: Tight glycemic control is essential for optimal outcomes after coronary artery bypass graft (CABG) surgery, regardless of pre-operative diabetes status. The ideal timing for transitioning from intravenous (IV) to subcutaneous (SC) insulin remains unclear. This study addresses this knowledge gap by comparing the effects of early transition (postoperative day 1, POD1) versus delayed transition on glycemic control and patient outcomes after CABG surgery. Methods: We analyzed data from a single tertiary medical center focusing on patients receiving insulin during their CABG hospitalization between 1 and 31 October 2022. We divided patients into two groups based on their transition timing: (1) Delayed Transition Group, patients transitioned from IV insulin infusion to SC insulin after POD1; and (2) Early Transition Group, patients transitioned on POD1. The primary outcome was the incidence of euglycemia on POD1. Secondary outcomes included rates of maintaining euglycemia from POD1 until POD10 or hospital discharge, hospital length of stay (LOS), ICU LOS, mean glucose levels, rates of hyperglycemia (blood glucose > 180 mg/dL) and hypoglycemia (blood glucose < 70 mg/dL), and rate of restarting IV insulin. Statistical analysis adjusted for BMI and diabetes diagnosis. Results: A total of 394 patients were enrolled, with 68 patients (17.3%) in the delayed-transition group and 326 patients (82.7%) in the early-transition group. Majority of the patients were males (74%), with an average age of 67 ± 9 years. Mean HbA1C and creatinine levels were comparable between the two groups. Patients in the early-transition group experienced a shorter ICU and hospital length of stay compared to the delayed-transition group, without a higher risk of restarting IV insulin. Conclusions: Early transition from IV insulin drip to SC insulin on POD1 of CABG surgery reduces ICU and hospital LOS without increasing the risk of transitioning back to IV insulin.

## 1. Introduction

Blood sugar control is an important aspect of caring for diabetic patients admitted to the intensive care unit (ICU). Studies have been conducted to compare the outcomes of tight versus liberal blood sugar control on patients admitted to the ICU; the NICE-SUGAR trial concluded that tighter glucose control with a target glucose of 81–108 mg per deciliter increases mortality and risk of severe hypoglycemia when compared with a more liberal glucose control approach that treats hyperglycemia only when it reaches above 180 mg per deciliter [1].

Coronary artery bypass graft surgery (CABG) is the most commonly performed cardiac surgery worldwide [2]. A significant proportion of patients have diabetes, which is associated with worse outcomes [3,4]. For patients undergoing cardiac surgery, poor glycemic control is associated with higher in-hospital mortality and postoperative complications, regardless of the pre-operative diabetic status [5,6,7]. A class 1B recommendation by the Society of Thoracic Surgeons (STS) is the use of intravenous (IV) insulin to keep blood glucose levels < 180 mg/dL during the operation and for the first 24 h postoperative period for diabetic patients undergoing cardiac surgery [8]. However, there is a lack of high-quality evidence guiding postoperative glycemic control beyond the initial 24 h [8,9].

This knowledge gap creates challenges, as IV insulin administration and titration through a continuous infusion requires close monitoring. The transition from IV to subcutaneous (SC) insulin has insufficient guidance for postoperative patients [10]. This study investigates the impact of early transition (postoperative day 1, POD1) versus delayed transition from IV to SC insulin on glycemic control and clinical outcomes in CABG patients.

## 2. Materials and Methods

This study is a retrospective cohort analysis approved by our Institutional Review Board (IRB). It was conducted at a tertiary hospital with a dedicated cardiac ICU, where a standardized insulin protocol guides IV insulin infusion by nursing staff. This protocol mandates hourly blood glucose checks until the patient has three consecutive readings within the target range of 140–180 mg/dL. Once achieved, monitoring frequency is reduced to every two hours. Patients are transitioned to SC insulin when deemed clinically appropriate by the attending physician in collaboration with a multidisciplinary team of physicians, nurses, nutritionists, and pharmacists during multidisciplinary rounds.

### 2.1. Population

Adult patients undergoing CABG surgery at our institution between 1 January 2019 and 31 October 2022 were included if they received continuous insulin infusion during their hospital stay. We excluded patients who were pregnant, incarcerated, under 18 years old, had missing demographic information, lacked blood glucose measurements, or never achieved the target blood sugar range (70–180 mg/dL) as defined in our study, which aligns with the NICE-Sugar study’s upper limit of recommended blood glucose levels [10]. Patients were retrospectively assigned to two groups based on their postoperative glycemic control and the clinical decisions made by their healthcare providers. The “Early Transition” group included patients who achieved controlled blood glucose levels earlier and were transitioned to subcutaneous (SC) insulin within the first postoperative day. This transition was a reflection of the routine clinical practices at our institution during the study period. The “Standard Protocol” group consisted of patients who required extended intravenous (IV) insulin infusion to achieve glycemic control.

To ensure a more homogeneous study population and reduce potential confounding factors, specific exclusion criteria were applied. We excluded patients who did not have a known sex, patients who did not receive IV insulin or subcutaneous insulin, patients who received less than 24 h of insulin therapy, patients who were receiving oral hypoglycemic therapy while hospitalized, patients who had missing glucose lab values, patients who never reached euglycemia, and one patient was also excluded due to unknown length of stay.

### 2.2. Outcomes Studied

The primary outcome measure was the incidence of euglycemia, defined by the recommended blood glucose levels range of 70–180 mg/dL, on POD1. Other outcomes measured included rates of maintained euglycemia from POD1 until POD10 or hospital discharge, hospital length of stay (LOS), ICU LOS, and mean glucose values. Safety measures included the rates of hyperglycemia (blood glucose > 180 mg/dL) and hypoglycemia (blood glucose < 70 mg/dL), and changes in the treatment course included the rate of restarting IV insulin therapy.

### 2.3. Statistical Analysis

Descriptive statistics were utilized to summarize baseline characteristics and the frequency of hypoglycemic and hyperglycemic events. Chi-Square and Fisher’s Exact test were used for categorical data; for example, Fisher’s exact test was used to assess the difference in mortality between the two groups. Negative binomial regression was used to evaluate LOS due to its suitability for over dispersed count data. Logistic regression was used to assess the rate of restarting IV insulin. Subgroup analyses were performed for different BMI groups and between patients with diabetes versus non-diabetic patients. A *p* value of <0.05 was used to determine significance. A Shapiro–Wilk test was used to assess the normality of the quantitative variables, and they were found to be normally distributed.

Univariate testing for all variables was not performed, given that, statistically, these tests cannot account for unmeasured confounding beyond the two variables being tested, which can lead to incorrect model selection and multiple comparisons bias, further distorting variable selection. All variables were evaluated for model inclusion, and those selected maintained clinical and statistical relevance to the outcome. Data were analyzed using SAS software, Version 9.4 for Windows. Copyright © 2020 SAS Institute Inc. (Cary, NC, USA). Statistical analysis was performed by a trained statistician (RM).

### 2.4. Definitions

Our study investigated the transition from intravenous (IV) to subcutaneous (SC) insulin administration in hospitalized patients. Hyperglycemia was defined as two consecutive blood glucose readings exceeding 180 mg/dL, while hypoglycemia was a single reading below 70 mg/dL, reflecting the greater concern for hypoglycemia during hospitalization [9].

## 3. Results

**Figure 1:**
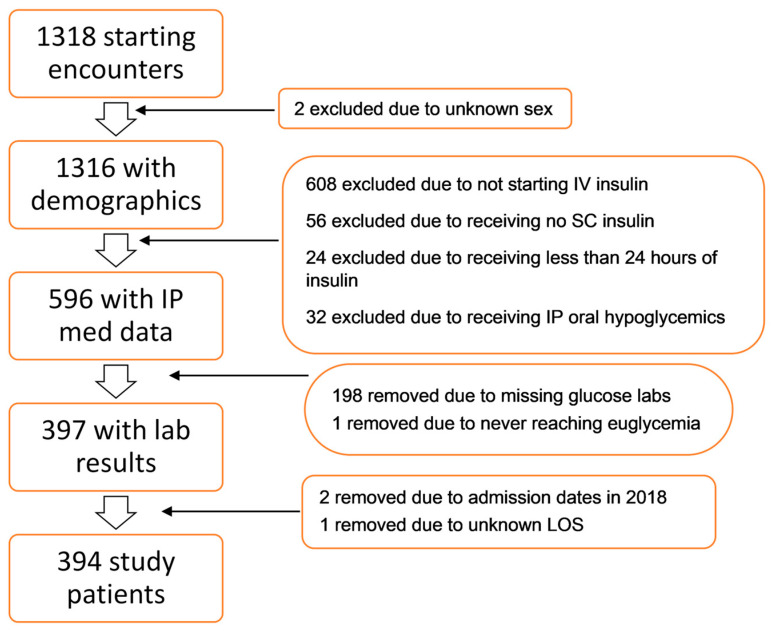
Inclusion and exclusion criteria. IP: inpatient, med: medications, LOS: length of stay.

In total, 1318 patients who underwent CABG were first identified for inclusion in the study. Two patients were excluded due to missing demographic information, and 720 patients were excluded for not receiving IV insulin after the surgery, not transitioning to SC insulin (either remaining on IV insulin or transitioning off insulin completely), receiving less than 24 h of insulin treatment, or receiving oral hypoglycemic agents. As a result, the initial sample consisted of 596 patients. Subsequent exclusions were made due to missing glucose levels (*n* = 198), failure to reach euglycemia (*n* = 2), admission outside the inclusion period (*n* = 2), and one patient with an unknown length of stay (LOS), resulting in a final sample size of 394 patients. A flowchart depicting the patient exclusions is presented in Figure 1.

### 3.1. Population Characteristics

We divided our patient cohort into two groups: delayed-transition group, i.e., patients who were not transitioned from IV to SC insulin on the day of or the day after the CABG surgery (Group 1, 68 patients [17.3%]), and early-transition group, patients who were transitioned from IV to SC insulin on the day of or the day after the CABG procedure (Group 2, 326 patients [82.7%]). The baseline characteristics of the patient cohort, stratified by group, are summarized in Table 1. This study included 394 patients, among whom 219 (55.6%) had diabetes. The proportion of patients with diabetes was similar in both groups (55.8% vs. 55.5%). Most patients were male (74%), with an average age of 67 years ± 9.

Regarding racial composition, the majority of the population was White (87%), while non-White patients constituted 13% of the sample, with a balanced distribution across the two groups. The mean hemoglobin A1C (HbA1C) level was 6.94 ± 1.81 for Group 1 and 6.71 ± 1.54 for Group 2 (*p* = 0.45). The mean creatinine level during the patient’s hospital stay was comparable between the two groups (1.18 ± 0.65). Similarly, the average glucose level on postoperative day one and throughout the hospital stay exhibited no significant differences between the two groups (Table 1).

In our cohort, we examined the relationship between the indications for CABG and the timing of postoperative glycemic transition. By analyzing the top three ICD-10 codes associated with CABG admissions, we identified valvular heart disease, acute coronary syndrome (ACS), and ischemic heart disease (IHD) with triple vessel involvement as the primary indications for the procedure.

Our findings are shown in Table 2.

### 3.2. Glycemic Control

Of the 394 patients, 91% (358) experienced hyperglycemia exceeding recommended levels. Conversely, 19% (74 patients) had hypoglycemic events, and 14.9% (59 patients) required a switch back to continuous IV insulin from SC administration. Of these 59 patients, 9 (13.2%) were in Group 1, and 50 (15.3%) were in Group 2.

Binary logistic regression was used to predict the likelihood of the return to IV insulin between the two groups and showed that early transition on day one to SC insulin is not associated with the possibility of returning to IV insulin (χ^2^ = 0.315, *p* = 0.57). However, a diagnosis of diabetes was a strong predictor, of returning to IV insulin infusion (OR = 10.389) [χ^2^ = 23.352, *p* < 0.001].

**Figure 2:**
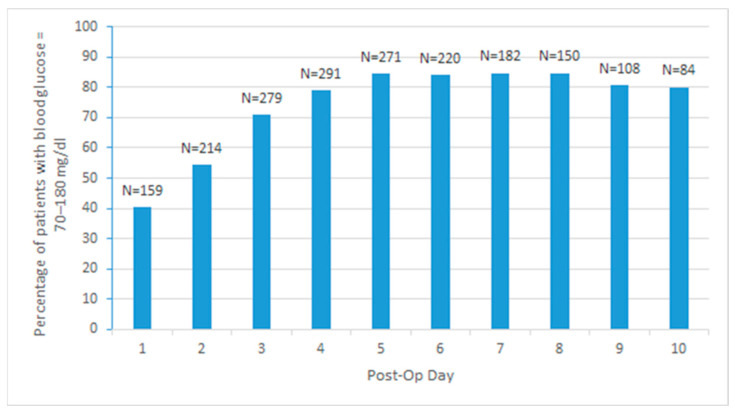
The percentage of patients who maintained blood glucose levels between 70 and 180 mg/dL daily (depicted on this Figure as a percentage of euglycemic patients). The percentage denominator is the number of patients admitted by that postoperative day, excluding patients discharged.

On the first postoperative day, 159 patients (40.4%) maintained euglycemia (blood glucose 70–180 mg/dL), 32 of these were in Group 1 (47%), and 127 (39%) were in Group 2. This number significantly increased to 46 patients for Group 1 (68%) and 233 patients (71%) for Group 2 by the third postoperative day (Figure 2), (Table 3 and Table 4).

Patients with normal body mass index (BMI) were more likely to achieve and maintain euglycemia compared to overweight (BMI 25–30 kg/m^2^) and obese (BMI ≥ 30 kg/m^2^) patients (Table 3).

Table 4 presents a profile of blood glucose levels, showing the number and percentage of patients from each group who were still hospitalized and achieved euglycemia at each postoperative day (POD).

Patients who remained on IV insulin on POD1 had a significantly higher probability of maintaining euglycemia than those who transitioned to SC insulin on POD1 (17.65% vs. 7.36%; χ^2^ = 7.332, *p* < 0.05).

The data presented in Table 4 illustrate the number and percentage of euglycemic patients from POD1 to POD10 in both the delayed- and early-transition groups. On POD1, a higher percentage of patients in the delayed-transition group (Group 1) achieved euglycemia compared to the early-transition group (Group 2). This trend continued on POD2.

However, by POD3, the trend reversed, with a lower percentage of patients in Group 1 achieving euglycemia compared to Group 2. Despite these variations, the differences in euglycemia rates between the two groups were not statistically significant on any of these days (POD1: *p* = 0.3; POD2: *p* = 0.7; POD3: *p* = 0.6).

### 3.3. Transition from IV to SC Insulin During POD1 and the Effect on LOS and Mortality

Negative binomial regression analysis showed that early transitioning from IV to SC insulin by postoperative day one is associated with a decreased length of hospital stay by a factor of 0.776 when compared with not transitioning to SC insulin by day 1 [χ^2^ = 12.22, *p* < 0.001, 95% CI (0.674, 0.895)]. Similarly, early transitioning by postoperative day 1 was associated with a decreased ICU LOS by a factor of 0.747 [χ^2^ = 17.6, *p* < 0.001, 95% CI (0.652, 0.856)]. The results show that delaying the transition to SC insulin was associated with a 30% (RR 1.3) increase in the count of days admitted to the hospital. Age and sex were included as control variables in all analyses. Furthermore, analysis with Fisher’s exact test revealed no significant difference in mortality rates between early-transition and delayed-transition groups (*p* = 1.0). Age and sex were controlled for in all analyses. Table 5 summarizes this study’s outcomes.

## 4. Discussion

We investigated the optimal timing of transitioning from IV to SC insulin after CABG surgery, comparing individuals who transitioned to SC insulin after day POD 1 (Group 1) to those who transitioned early (Group 2). We found that 40.4% of patients achieved euglycemia on POD1, with an average glucose of 164 mg/dL in both groups, demonstrating fair glycemic control. However, our results revealed a significant trade-off between the timing of the transition to SC insulin and patient outcomes. Specifically, patients who were transitioned to SC insulin on POD1 (early transition) experienced shorter ICU and hospital stays, which may be clinically advantageous, as prolonged hospitalization can increase the risk of hospital-acquired complications and increase healthcare costs. In contrast, the delayed-transition group, while experiencing fewer hyperglycemic episodes, had a longer hospital stay.

This paradox can be attributed to the more intensive glucose monitoring and tighter control required during the prolonged use of an insulin drip, which necessitates a longer period of stability before transitioning to SC insulin. The extended length of stay (LOS) associated with delayed transition underscores the importance of balancing glycemic control with the risks associated with prolonged hospitalization. Our findings suggest that while a delayed transition may reduce episodes of hyperglycemia, early transition may offer a more balanced approach by minimizing LOS without compromising glycemic stability. Further research is needed to explore these dynamics and optimize glycemic management strategies post-CABG, with a focus on both clinical outcomes and resource utilization.

While the delayed-transition group (Group 1) initially exhibited slightly better glycemic control, the early-transition group (Group 2) caught up by POD3, achieving higher rates of euglycemia. However, the lack of statistical significance indicates that the timing of the transition to SC insulin—whether early or delayed—does not lead to a meaningful difference in glycemic control. This supports the clinical viability of both approaches, allowing for flexibility based on individual patient needs without compromising glycemic outcomes.

Cardiac surgery patients, especially those undergoing CABG, are at high risk for stress hyperglycemia and associated adverse effects [6,8,11]. In addition, multiple studies have shown the importance of glycemic control in hospitalized patients [9,11,12]. The STS recommends continuing an IV insulin at least 24 h postoperatively in the ICU, and it is a common practice to continue IV insulin for three days postoperatively as the longer duration of IV insulin infusion allows for a faster correction of aberrant glucose values to combat the untoward effects of hyper and hypoglycemia. However, the evidence supporting this extended IV insulin duration is limited [8,11,13,14].

Glycemic control management in CABG patients is challenging [15]. Strong evidence favors more conservative glucose targets than aggressive control, citing fewer hypoglycemic events and improved outcomes [9,16]. Other studies have shown average glucose between 150 and 160 mg/dL to be as effective as maintaining lower average glucose levels [17,18]. Our cohort achieved this glucose control level even though most patients transitioned to an SC regimen on POD1.

Previous studies have indicated that switching to SC insulin before the three-day mark may be as effective in achieving euglycemia as continuing IV insulin infusion. Schmeltz et al. investigated glucose control in cardiothoracic surgery patients utilizing a combination of IV insulin infusion in the ICU and SC insulin outside the ICU [19]. This switch to SC insulin was made without completing three days of IV insulin infusion. Similar to our study, Schmeltz et al. found that three days of IV insulin infusion was not necessary for adequate glucose control. More specifically, they found that utilizing IV and SC insulin eliminates the increase in mortality for diabetic patients in a subgroup of patients undergoing CABG, as well as in other cardiothoracic surgeries [19]. Based on these results, the authors concluded their combined approach was less costly and less taxing on nursing staff than continuing IV insulin for three days.

Stahnke et al. transitioned open-heart-surgery candidates undergoing CABG or valve replacement from IV to SC insulin for [20]. Similar to our study, most of their patients were transitioned to SC insulin before POD2. Their study examined the implementation of a new transition protocol to achieve better glucose control, and while the average glucose on POD1 and POD2 were not significantly different between the groups, the post-protocol group experienced substantially less hypoglycemia, and less than 10% of patients had blood glucose levels ≥ 200 mg/dL on POD2.

Glucose control improved over time in our study, as would be expected. While 40.4% of patients were considered euglycemic on POD1, this number increased to 70.8% on day three and 84.4% on day 5. Many patients remained euglycemic from day three onward, indicating clinical stability and adequate glycemic control.

The average hospital LOS was 8.1 days in the group that transitioned early to SC insulin on POD1 compared to 10.4 days in the group with delayed transition. The LOS for the delayed-transition group is similar to that of patients in the Stahnke study [20]. Similarly, ICU LOS was 7.2 days on average in our early-transition group and longer, 9.7 days, in the delayed-transition group. These findings were statistically significant. In the interpretation of these findings, it is important to note that factors such as the patient’s continued dependence on invasive mechanical ventilation or the use of vasoactive agents were not controlled. Additionally, we acknowledge that both the length of hospital stay (LOS) and ICU LOS are multifactorial outcomes influenced by a range of variables. These include the initial indications for CABG, the complexity and outcomes of the surgical procedures, patient comorbidities, and perioperative complications. Future studies should aim to comprehensively evaluate these factors to provide a more nuanced understanding of their impact on LOS and ICU LOS. This consideration is essential for developing holistic postoperative management strategies. However, from a qualitative standpoint, the data suggest a plausible safety profile for the early transition to SC insulin in a substantial subset of patients.

Unsurprisingly, euglycemia rates decreased with increasing BMI. This is likely explained by greater insulin resistance in the group with higher BMI. This emphasizes the importance of individualizing insulin regimen for CABG patients.

Another noteworthy finding was the difference in the frequency of restarting continuous IV insulin infusion from an SC insulin regimen. Overall, return to IV insulin infusion was uncommon, with only 15% of our cohort requiring an IV insulin infusion after transitioning to SC insulin. However, patients with diabetes were over ten times as likely to return to IV insulin drip in our study. This shows, yet again, that diabetes as a comorbid condition is a crucial factor to consider. However, while the transition back to IV insulin drip was likely due to hyperglycemia, there are potentially other reasons a patient may have transitioned back to IV insulin that could not be specified given this study’s limitations.

Finally, hyperglycemia was common in our study, as is often the case in CABG patients. In total, 90.9% of our cohort experienced blood sugar levels > 180 with an average of three events per patient, bolstering the argument that glucose control is imperative. On the other hand, hypoglycemia was not as common in our cohort. Specifically, 18.8% of patients experienced at least one glucose reading of <70 mg/dL.

Our study has several limitations due to its retrospective design. Firstly, there was a significant difference in sample sizes between the two groups. This discrepancy likely results from the fact that insulin infusion is less likely to be required after the first postoperative day (POD1) for glycemic control, leading to an earlier transition to subcutaneous (SC) insulin. Studies support this observation, indicating that IV insulin infusion is typically not needed for extended periods to manage glycemic status. Protocols often recommend transitioning to SC insulin once blood glucose levels stabilize and infusion rates are steady, usually within 24 h of starting the infusion [19]. Additionally, as shown in Table 1, the baseline characteristics of the two groups, including sex, age, race, pre-existing diabetes diagnosis, and BMI distribution, did not show any significant differences, ensuring the validity of comparisons between the groups despite the differences in sample sizes.

Secondly, the retrospective nature of our study limits our ability to directly correlate surgical outcomes and indications for surgery with glycemic control, highlighting the need for further prospective studies to explore this relationship.

Furthermore, key baseline data, including the classification of patients as Type I or Type II diabetes, the specific baseline treatment regimens (non-insulin medications, insulin, or no treatment), the duration of diabetes prior to the intervention, and existing diabetic complications (such as vasculopathy, neuropathy, or retinopathy), were not available for individual patients. Consequently, we are unable to assess how these factors might differ between groups or their potential influence on the transition from IV to SC insulin. The lack of this information may introduce bias into our results. Further studies are needed to take these factors into account.

While our study highlights the significance of blood glucose management in influencing postoperative outcomes, it is important to note that LOS and ICU LOS are affected by multiple factors beyond the scope of our analysis. Our inability to stratify patients based on these variables is a limitation that future research should address to provide a more comprehensive understanding of the factors influencing LOS and ICU LOS.

It is important to note that we cannot establish causation from observed associations. We attempted to mitigate confounding by adjusting for known factors like BMI and diabetes diagnosis. Additionally, there was no standardized protocol to determine insulin requirements for patients transitioning from IV to SC. There is also no way to tell why the comparator group was kept on IV insulin beyond POD1, and understanding why some patients in the delayed-transition group remained on IV insulin beyond POD1 would be helpful in refining future protocols. For the patients who were transitioned back from SC to IV insulin, the timing of the transition could not be specified, which adds to the limitations of our study.

Furthermore, while blood glucose levels provide valuable information, they are an indirect measure of overall glycemic control. While we tried to investigate some important clinical outcomes like sternal wound infection and long-term mortality, the sample size and follow-up period were likely to miss capturing these events adequately. Finally, we did not control for NPO status, or mechanical ventilation, which could introduce collinearity with the primary predictor (transition timing). These factors likely influence the decision to transition to SC insulin, potentially masking the true effect of transition timing on outcomes.

Prospective studies with standardized protocols and a larger sample size could definitively assess the safety and efficacy of early transition to SC insulin for specific patient profiles. Furthermore, longer follow-up periods would be valuable for evaluating long-term outcomes.

## 5. Conclusions

Our study shows that early transition from IV insulin infusion to SC insulin after CABG appears safe and is associated with shorter hospital and ICU length of stays. Although the group transitioned to SC insulin later than POD1 had fewer hyperglycemic episodes, there were no differences in the likelihood of reverting to IV insulin between the groups. The average glucose values between the groups were numerically similar. Future prospective trials with standardized protocols are needed to optimize timing and protocols for transitioning to SC insulin after CABG surgery.

## Figures and Tables

**Table 1:** Demographics and baseline characteristics in each group.

Variable	Level	Total	Group 1 (Delayed Transition to SC Insulin)	Group 2 (Early Transition to SC * Insulin)	*p*-Value
N (%)		394 (100.00)	68 (17.26)	326 (82.74)	
Sex, N (%)	Male	102 (25.89)	18 (26.47)	84 (25.77)	0.88
	Female	292 (74.11)	50 (73.53)	242 (74.23)	
Race, N (%)	Non-White	50 (12.69)	9 (13.24)	41 (12.58)	0.88
	White	344 (87.31)	59 (86.76)	285 (87.42)	
Diabetes, N (%)	Yes	175 (44.42)	30 (44.12)	145 (44.48)	0.96
	No	219 (55.58)	38 (55.88)	181 (55.52)	
Age years, M (SD)		67.34 (9.15)	66.79 (9.34)	67.45 (9.12)	0.7
BMI kg/m^2^, M (SD)		31.44 (5.97)	32.25 (6.89)	31.27 (5.75)	0.32
Creatinine mg/dL, M (SD)		1.18 (0.65)	1.19 (0.50)	1.18 (0.68)	0.884
HbA1C ** %, M (SD)		6.75 (1.59)	6.94 (1.81)	6.71 (1.54)	0.45

Group 1: (delayed transition to SC insulin) patients who were not transitioned from IV to SC insulin on the day of or the day after (POD1) the CABG surgery. Group 2: (early transition to SC insulin) patients who were transitioned from IV to SC insulin on POD1. * SC: subcutaneous. ** 64 patients missing HbA1C values–11 in Group 1, 53 in Group 2. N: number, M: mean, SD: standard deviation, BMI: body mass index.

**Table 2:** Timing of postoperative glycemic transition by indication for CABG.

Indication for CABG	Total N of Cases	Transitioned by Postoperative Day 1. N (%)	Transitioned Later. N (%)
Valvular Heart Disease	40	35 (87.5%)	5 (12.5%)
Acute Coronary Syndrome (ACS)	133	90 (67.7%)	43 (32.3%)
Ischemic Heart Disease with Triple Vessel Involvement	221	201 (91%)	20 (9%)

N: number; %: percentage.

**Table 3:** Patients maintaining blood glucose levels of 70–180 at day 1 through 10 by BMI group.

	Total			Normal BMI **		Overweight		Obese		Severely Obese	
Day	N D/C *	N	%	N	%	N	%	N	%	N	%
1	0	159	40.36	22	61.11	66	43.42	63	37.06	8	22.22
2	0	214	54.31	23	63.89	90	59.21	90	52.94	11	30.56
3	0	279	70.81	28	77.78	114	75	121	71.18	16	44.44
4	25	291	78.86	26	78.79	111	79.29	132	81.99	22	62.86
5	73	271	84.42	22	78.57	104	85.95	120	86.33	25	75.76
6	132	220	83.97	18	75	83	86.46	95	84.82	24	80
7	179	182	84.65	14	73.68	68	86.08	80	86.96	20	80
8	217	150	84.75	12	75	57	86.36	63	87.5	18	78.26
9	260	108	80.6	10	71.43	46	83.64	40	83.33	12	70.59
10	289	84	80	6	60	38	84.44	35	87.5	5	50

* N D/C: Cumulative number of patients discharged by this day. ** BMI: body mass index. Normal BMI: 18.5 to 24.9, overweight: BMI between 25 and 29.9, obese: BMI between 30 and 34.9, severely obese: BMI > 35.

**Table 4:** Number of patients discharged from each group for each day and the number and percentage of euglycemic patients at days 1 through 10 for each group.

Day	Total Patients Discharged	Number of Group 1 Patients Who Were Discharged	Number of Group 2 Patients Who Were Discharged	Total Euglycemic Patients N (%)	Group 1 Euglycemic Patients N (%)	Group 2 Euglycemic Patients N (%)
1	0	0	0	159 (40.36)	32 (47.06)	127 (38.96)
2	0	0	0	214 (54.31)	39 (57.35)	175 (53.68)
3	0	0	0	279 (70.81)	46 (67.65)	233 (71.47)
4	25	1	24	291 (78.86)	52 (77.61)	302 (79.14)
5	73	6	67	271 (84.42)	51 (82.26)	220 (84.94)
6	132	13	119	220 (83.97)	45 (81.82)	175 (84.54)
7	179	21	158	182 (84.65)	38 (80.85)	144 (85.71)
8	217	29	188	150 (84.75)	30 (76.92)	120 (86.96)
9	260	38	222	108 (80.60)	21 (70.00)	87 (83.35)
10	289	45	244	84 (80.00)	17 (73.91)	67 (81.71)

Group 1: (delayed transition to SC Insulin) patients who were not transitioned from IV to SC insulin on the day of or the day after (POD1) the CABG surgery. Group 2: (early transition to SC insulin) patients who were transitioned from IV to SC insulin on POD1.

**Table 5:** Study outcomes for each group.

Outcome	Total	Group 1 (Delayed Transition to SC Insulin)	Group 2 (Early Transition to SC Insulin)	*p*-Value
LOS days, M (SD)	8.47 (5.78)	10.43 (7.19)	8.06 (5.36)	<0.001
ICU LOS days, M (SD)	7.65 (5.14)	9.74 (6.75)	7.22 (4.64)	<0.001
Day1 Avg Glucose mg/dL, M (SD)	164.06 (13.69)	163.82 (19.55)	164.11 (12.16)	0.9
Postop Avg Glucose mg/dL, M (SD)	158.19 (15.20)	155.28 (15.33)	158.80 (15.13)	0.19
Maintenance of Euglycemia, N (%)	98.54 (25.01)	69.5 (17.65)	29 (7.36)	<0.05
Mortality, N (%)	5 (1.27)	1 (1.47)	4 (1.23)	1.000

Postop Avg Glucose: average glucose level from day of CABG procedure to discharge. Maintenance of Euglycemia: in our study, we defined this by maintenance of a blood glucose level between 70 and 180 mg per deciliter.

## Data Availability

Data supporting the reported results is provided in the article. Additional deidentified data are available to the first author and the statistical analyst R.M.

## Abbreviations

The following abbreviations are used in this manuscript:

BMI	Body mass index
CABG	Coronary artery bypass graft surgery
HbA1C	Hemoglobin A1C
ICU	Intensive care unit
IRB	Institutional Review Board
LOS	Length of stay
NPO	Nothing by mouth
POD1	Postoperative day 1
SC	Subcutaneous
STS	Society of Thoracic Surgeons
